# Metabolic syndrome and aberrant immune responses to viral infection and vaccination: Insights from small animal models

**DOI:** 10.3389/fimmu.2022.1015563

**Published:** 2022-11-30

**Authors:** Elizabeth Geerling, Muddassar Hameed, James Weger-Lucarelli, Amelia K. Pinto

**Affiliations:** ^1^ Department of Molecular Microbiology and Immunology, Saint Louis University School of Medicine, St. Louis, MO, United States; ^2^ Department of Biomedical Science and Pathobiology, Virginia-Maryland College of Veterinary Medicine, Virginia Polytechnic Institute and State University, Blacksburg, VA, United States; ^3^ Center for Zoonotic and Arthropod-borne Pathogens, Virginia Polytechnic Institute and State University, Blacksburg, VA, United States

**Keywords:** metabolic syndrome, obesity, type 2 diabetes, hypertension, dyslipidemia, vaccination, vaccine efficacy, viral infection

## Abstract

This review outlines the propensity for metabolic syndrome (MetS) to induce elevated disease severity, higher mortality rates post-infection, and poor vaccination outcomes for viral pathogens. MetS is a cluster of conditions including high blood glucose, an increase in circulating low-density lipoproteins and triglycerides, abdominal obesity, and elevated blood pressure which often overlap in their occurrence. MetS diagnoses are on the rise, as reported cases have increased by greater than 35% since 1988, resulting in one-third of United States adults currently diagnosed as MetS patients. In the aftermath of the 2009 H1N1 pandemic, a link between MetS and disease severity was established. Since then, numerous studies have been conducted to illuminate the impact of MetS on enhancing virally induced morbidity and dysregulation of the host immune response. These correlative studies have emphasized the need for elucidating the mechanisms by which these alterations occur, and animal studies conducted as early as the 1940s have linked the conditions associated with MetS with enhanced viral disease severity and poor vaccine outcomes. In this review, we provide an overview of the importance of considering overall metabolic health in terms of cholesterolemia, glycemia, triglyceridemia, insulin and other metabolic molecules, along with blood pressure levels and obesity when studying the impact of metabolism-related malignancies on immune function. We highlight the novel insights that small animal models have provided for MetS-associated immune dysfunction following viral infection. Such animal models of aberrant metabolism have paved the way for our current understanding of MetS and its impact on viral disease severity, dysregulated immune responses to viral pathogens, poor vaccination outcomes, and contributions to the emergence of viral variants.

## Introduction

**Figure 1:**
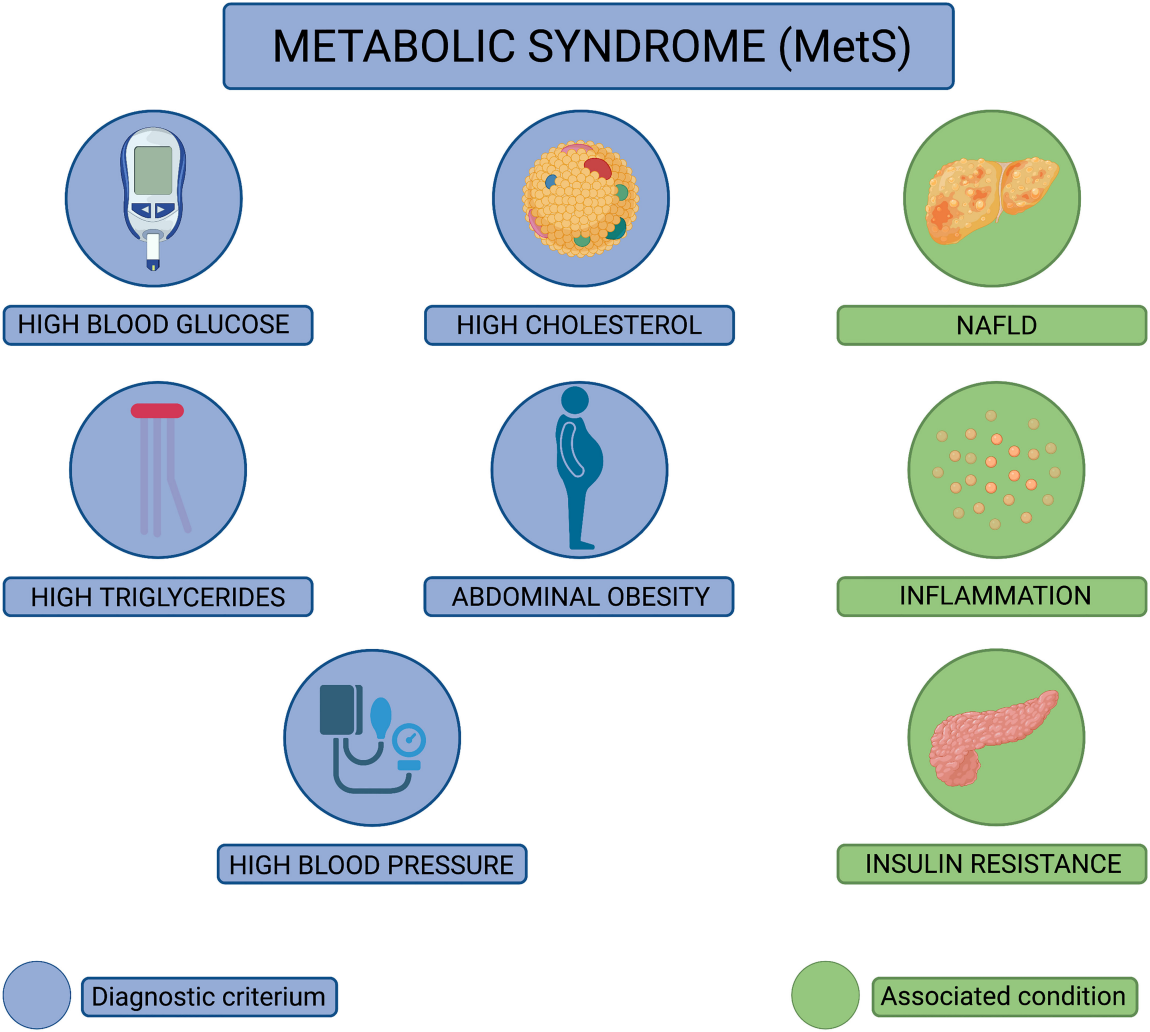
Diagnostic criteria for metabolic syndrome (MetS). Pictorial representation of the diagnostic criteria used to diagnose metabolic syndrome (MetS). MetS is diagnosed when an individual displays at least three of the following pathophysiological conditions: high blood glucose, high cholesterol, high triglycerides, abdominal obesity, and high blood pressure (conditions depicted in blue). In addition to these conditions, MetS patients often experience nonalcoholic fatty liver disease (NAFLD), chronic inflammation, and insulin resistance (conditions depicted in green).

First described in 1977 (1), metabolic syndrome (MetS) is diagnosed in an individual whose metabolism is disrupted, leading to an imbalance in the processing of food for energy, the synthesis of protein, lipids, and amino acids, as well as the elimination of metabolic waste. Alarmingly, MetS diagnoses have increased by 35% since 1988, culminating in one-third of American adults diagnosed with MetS (2). The diagnostic criteria for MetS are when an individual concurrently experiences three or more of the following conditions: high blood glucose, high levels of circulating low-density lipoprotein, high levels of circulating triglycerides, abdominal obesity, and high blood pressure (2–4) (**Figure 1**). As such, it is very common for these subcomponents of MetS to occur concurrently within a patient. MetS patients also often experience insulin resistance and nonalcoholic fatty liver disease, with evidence implicating chronic inflammation as the link between the MetS diagnostic criteria (5). Underlying causes of MetS are multifactorial, including being overweight or obese, resistant to insulin, having a sedentary lifestyle, predisposing genetic factors, and advanced age (2). While it has been well established that MetS enhances the risk for developing life-threatening conditions including heart disease, type 2 diabetes, stroke, and is a risk factor for sudden cardiac death (3, 4, 6), the realization that individuals with MetS experience more severe disease following viral infections and reduced protection from vaccination have only recently been appreciated.

**Figure 2:**
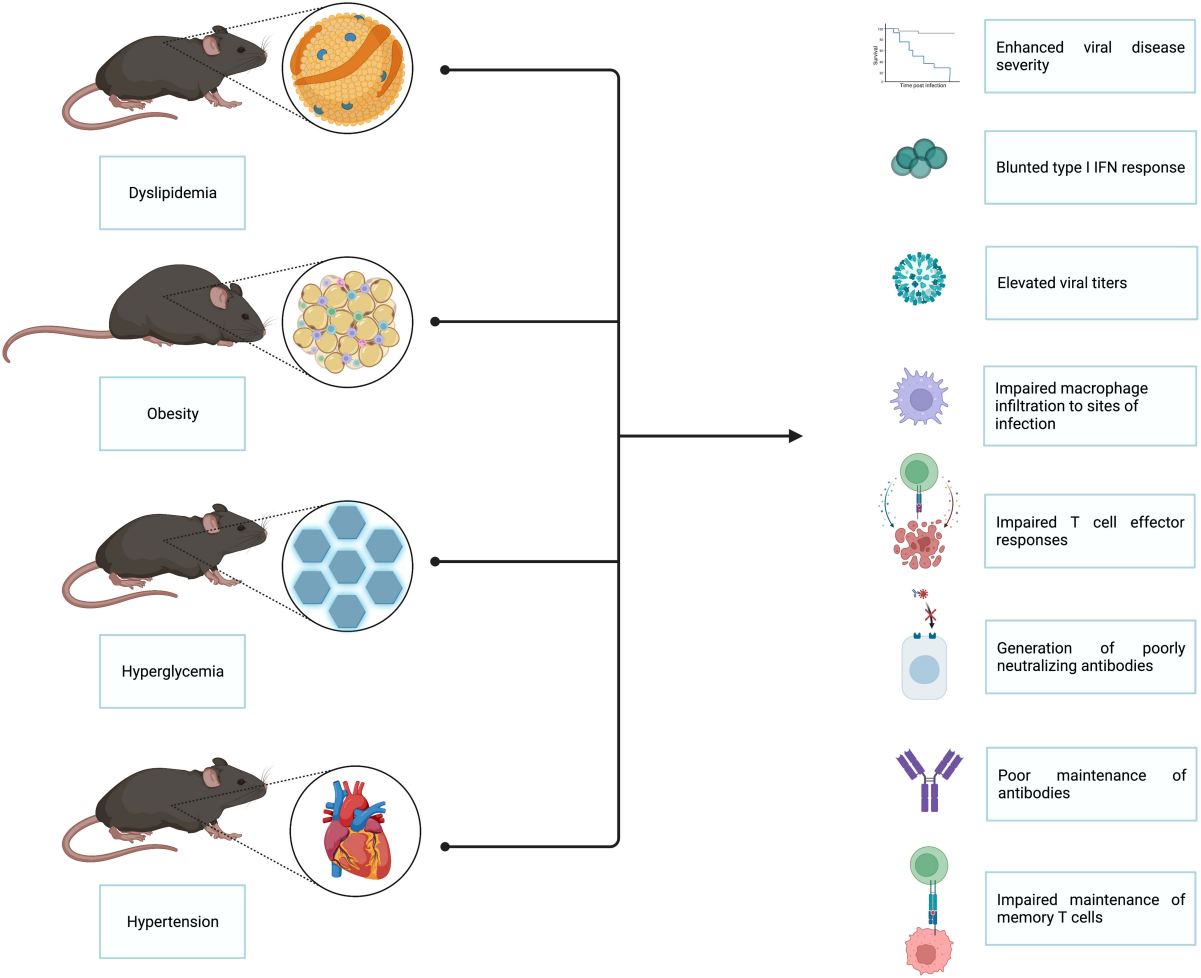
Insights gained from small animal models of the impact of MetS on viral immunity. Several small animal models have been utilized to interrogate the impact of MetS on viral immunity. The main animal models employed thus far are those that model dyslipidemia, obesity, hyperglycemia, and hypertension. Studies done utilizing these animal models have revealed that MetS-associated conditions lead to enhanced viral disease severity, blunted type I interferon responses, elevated viral titers, impaired macrophage infiltration to sites of infection, impaired T cell effector responses, generation of poorly neutralizing antibodies, poor antibody maintenance, and impaired maintenance of memory T cells.

In this review, we focus on the individual metabolic perturbances encompassed by MetS and highlight the animal studies which identify these conditions as predictors of elevated viral disease severity (7, 8), higher mortality rates following infection (9–11), and poor vaccination outcomes (12–15). Independently, each of the conditions associated with MetS is a risk factor for severe pathology; however, when these conditions present together, as they do in patients with MetS, the chance of developing serious physiological complications significantly increases (2, 6). While retrospective human cohort studies have laid much of the groundwork for linking metabolic perturbances to impaired viral immunity, small animal models serve as critical tools for understanding this phenomenon and uncovering the mechanisms driving dysregulated metabolism-associated immune dysfunction in response to viral pathogens. Throughout this review, we comment on works that utilized small animal models to explore the impact of high cholesterol, triglycerides, glucose, and hypertension on host immune responses to viral pathogens. Given that the different comorbidities associated with MetS often overlap in the types of immune dysfunction they induce, we have combined these known defects into one summarizing graphic, depicted in **Figure 2**. In addition, we summarize how such metabolic perturbances have been shown to enhance viral disease severity and influence the emergence of virulent viral variants.

## Impact of high cholesterol and triglyceride levels on viral immunity

As two diagnostic criteria for MetS diagnosis, elevated cholesterol and triglyceride levels have long been shown to influence the susceptibility to viral infection and disease severity. Cholesterol is a sterol synthesized by all animal cells, essential for providing structural integrity to cells and as a building component for vitamins and hormones (16). Cholesterol travels through the body inside of lipoproteins which are comprised of fat and protein. There are two major types of cholesterol-carrying lipoproteins: high-density lipoprotein (HDL) and low-density lipoprotein (LDL). LDL, often known as the bad cholesterol, contributes to fat deposition within arteries (17). Conversely, HDL transports cholesterol away from the arteries to the liver where it can be metabolized and excreted from the body (18). Triglycerides are the most commonly found fat in the human body and are important for storing excess energy obtained through diet (18). When cholesterol and/or triglyceride levels fall outside of the normal range, the resulting phenomenon is referred to as dyslipidemia, which is associated with all-cause mortality and enhanced risk for cardiovascular disease (19). Due to the reliance of viruses on lipids to replicate and produce viral progeny, whether host dyslipidemia impacts viral infection outcomes is an exciting avenue for discussion.

It is well understood that viruses hijack host lipid metabolism by manipulating gene expression to sustain their lifecycles and produce new progeny virions (reviewed in (20)). Studies done with viruses such as influenza A virus (IAV), herpes viruses, and hepatitis viruses have eloquently highlighted the significant alterations in lipid metabolism that occur within hosts following infection (21–26). Remarkably, these patterns of altered metabolism can be sustained even after viral clearance (27). Specifically, severe fever with thrombocytopenia syndrome virus (SFTSV) relies on host cell cholesterol, fatty acid, and triglyceride synthesis pathways to replicate and produce progeny; in fact, treating cells with inhibitors of these synthesis pathways before SFTSV infection significantly lowers the titer of infectious progeny post-infection (28). Previous work supports this idea as disruption of lipid rafts, lipid droplets, or diminished levels of circulating triglycerides can reduce the production of infectious rotavirus progeny (29–31) and interrupts hepatitis C (32, 33) and dengue virus replication (34, 35). These studies suggest that altering host lipid metabolism may be a mechanism to alter viral infection and reduce disease severity.

Multiple animal studies have supported clinical observations demonstrating the impact of high cholesterol on viral replication. Braunwald et al. employed a high cholesterol diet in a murine model of viral infection using A/J mice genetically modified not to be susceptible to mouse hepatitis virus type 3 (MHV3). The authors noted that following a hypercholesterolemic diet, these mice succumbed to MHV3 infection and had high MHV3 titers in their livers accompanied by necrotic hepatocytes and elevated serum transaminase levels (36), indicating intense virus-induced liver pathology in mice with high cholesterol levels. Delving deeper into the impact of dyslipidemia on susceptibility to viral infections, Loria et al. conducted a seminal study in which mice fed a diet rich in cholesterol were infected with coxsackievirus B. Following infection, the authors noted that mice with elevated cholesterol levels displayed enhanced morbidity to coxsackievirus B infection compared to mice with normal cholesterol levels (37). While these studies employed the use of high cholesterol diets to interrogate the impact of dyslipidemia on virus-induced morbidity, it is important to note that these diets were high in sucrose, making the results indicative of a positive correlation between unhealthy diet and enhanced severity of viral disease. Future studies using fine-tuned diets to induce hypercholesterolemia independent of heightened sucrose could bolster the reported relationship between hypercholesterolemia and enhanced viral disease severity.

**Figure 3:**
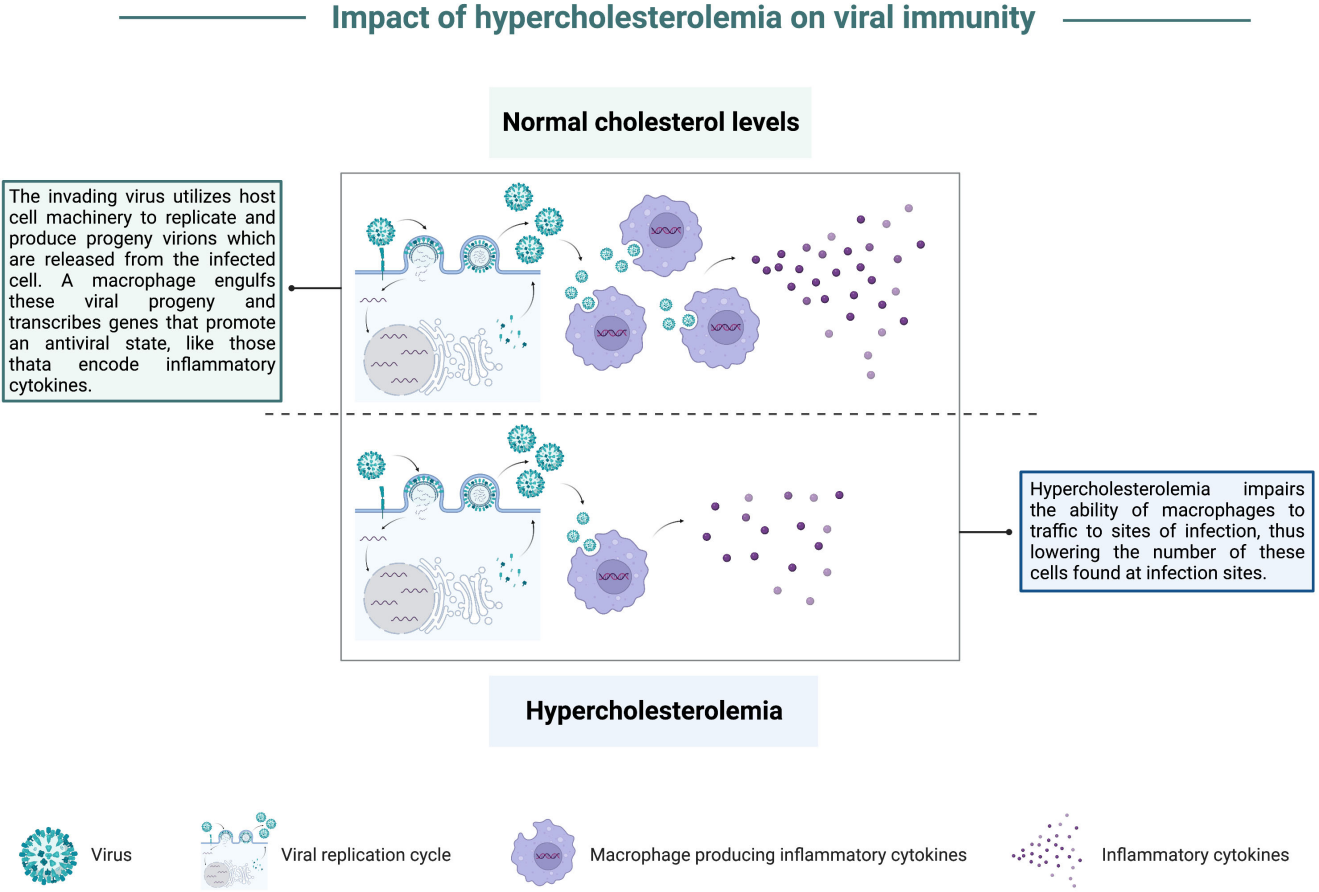
Impact of hypercholesterolemia on viral immunity. In a state of normal cholesterol levels, an invading virus hijacks host cell machinery to replicate and produce progeny virions that are released from the infected cell. A macrophage recruited to the infection site can engulf viral progeny and contribute to the anti-viral state by transcribing genes that stimulate the innate immune response, like pro-inflammatory cytokine-encoding genes. In a state of hypercholesterolemia, there is a defect in macrophage recruitment to infection sites. Consequently, there are fewer macrophages found at infection sites, blunting this arm of the immune response.

Combining the *in vitro* studies highlighting the importance of lipid metabolism for viral replication with *in vivo* animal models which mimic the impact of high cholesterol on viral infection, multiple studies have focused on the mechanisms to explain how elevated cholesterol and triglycerides contribute to increased viral disease severity. Campbell et al. found a correlation between high cholesterol and increased mortality in mice following coxsackievirus B infection, in addition to elevated viral titers in the blood and liver. These authors also noted that mice fed a cholesterol-rich diet had a defect in the ability of monocytes and macrophages to infiltrate into infection sites compared to the migration abilities of phagocytes primed in mice with normal cholesterol levels (38), graphically depicted in **Figure 3**. Utilizing a similar approach where the effects of a cholesterol-rich diet were compared with those of a standard diet, high cholesterol feeding resulted in dyslipidemia prior to IAV infection (39). Louie et al. reported that these high cholesterol mice displayed exacerbated morbidity yet did not show higher viral loads compared to mice with normal cholesterol levels post-IAV infection (39). Transcriptomics from lungs of mice fed a high cholesterol diet revealed an upregulation in the expression of genes involved in cytokine production by CD4 T, CD8 T, and dendritic cells (39). Furthermore, morbidity was also correlated with the numbers of cytokine-producing lymphocytes and granulocytes (39). These results suggest that high cholesterol levels enhance morbidity by exaggerating cellular immune responses. These findings bolster the idea that supraphysiological levels of cholesterol can contribute to worsened disease development following viral infection, and that lowering cholesterol levels can reduce the severity of virally induced disease in the host.

Given the evidence that cholesterol and triglycerides are important for modulating viral infection and disease severity, research efforts to modify lipid metabolism in animal models to improve infection outcomes have begun. For example, Karlsson et al. noted that obese mice with high cholesterol levels treated with a statin following IAV infection showed protection from severe viral disease. Yet, treatment of wild-type mice with a statin did not protect against severe viral disease (40). Multiple studies have demonstrated that statin treatment and lowered cholesterol levels have anti-inflammatory effects which could improve disease outcomes (reviewed in (41)). Further, other studies support a role for statins having a direct anti-viral effect based on *in vitro* findings that showed a significant reduction in Zika or dengue virus viral titers in cells treated with statins compared to controls (42, 43).

Based on findings from murine models of high cholesterol, it is unsurprising that human cohort analyses conducted during the SARS-CoV-2 pandemic have revealed that many COVID-19 patients requiring hospitalization had a history of low HDL and high triglyceride levels before infection, with more severe cases being correlated with lower HDL and higher triglyceride levels at the time of infection (44). Other data reported from SARS-CoV-2 infections revealed a link between a history of generalized dyslipidemia to severe cases of COVID-19 (45). Interestingly, Lee et al. noted that sterol regulatory element-binding protein 2 (SREBP-2)-induced inflammatory responses were elevated in COVID-19 intensive care unit patients (46). SREBP-2 is essential for cholesterol biosynthesis, suggesting that high cholesterol levels contributed to the cytokine storm and ensuing pulmonary damage in these COVID-19 patients. Further, these authors utilized a murine model to test whether inhibiting SREBP-2 impacted sepsis outcomes and demonstrated that blocking SREBP-2 activation helped suppress cytokine storm, pulmonary damage, and promoted high survival rates (46). The studies above provide a strong link between dyslipidemia and severe viral infection outcomes, although specific mechanisms driving this phenomenon have yet to be elucidated. However, these data suggest that heightened cholesterol levels could foster high levels of viral replication within the host, thus providing the potential for more severe disease. In turn, elevated viral titers could contribute to inflammation-mediated tissue pathogenesis and incite exaggerated cellular responses, which could lead to enhanced immune-mediated pathology.

## Impact of obesity on viral immunity

Obesity, abnormal or excessive fat accumulation, is a diagnostic component of MetS. Obesity rates have tripled worldwide since 1975, with more than 4 million people dying yearly due to complications associated with this condition (47). Specifically, within the US, current statistical models project 50% of adults will have obesity by 2030 (48), and global obesity rates are projected to encompass 50% of adults by 2050 (49). In the 1950s and 1960, increased susceptibility to viral infection and severe viral diseases was noted in studies of obese animals and instances of overnutrition in humans (50, 51). In the subsequent years, many studies emphasized a connection between obesity and severe viral disease (reviewed in (52)). By the early 2000s, research efforts focused on exploring ties between obesity and immune regulation were centered on the impact of obesity-associated inflammation on insulin resistance (53–57). However, only following the 2009 H1N1 pandemic was obesity cited by the US Centers for Disease Control and Prevention (CDC) as a risk factor that could predict severe viral infection outcomes. During the 2009 H1N1 pandemic, a large proportion of H1N1 patients hospitalized due to severe disease or who succumbed to infection were obese (58). Further, amidst the SARS-CoV-2 pandemic, obesity was again cited as a risk factor for severe SARS-CoV-2-induced illness and has been correlated with higher mortality rates following SARS-CoV-2 infection (7, 8, 59, 60). Intense efforts have been focused on determining mechanisms underlying metabolic dysfunction caused by obesity (56, 57, 61, 62), but the exact mechanisms by which MetS induces immune dysregulation and more severe viral disease are unknown and are an active area of research. Small animal models have been essential for dissecting metabolic pathways and how their dysregulation in the obese state can impact immune responses to viral pathogens.

Several murine models exist for use in the interrogation of obesity and associated metabolic perturbances on overall health, including genetically obese and diet-induced obese (DIO) mice. Although genetically obese animals serve as excellent recapitulatory models of morbid obesity, the leptin (ob/ob or *Lep^ob^*) or leptin receptor (db/db or *Lepr^db^*) mutations that induce obesity in these models rarely occur in humans. Thus, the use of DIO mice more closely models human obesity as it supports examining the effects of chronic over-nutrition and the ensuing oxidative stress it exerts on the immune system. Nonetheless, DIO models largely recapitulate the same immune system perturbances as genetically obese models, but the resulting phenotypes in DIO mice tend to be less exaggerated. In this section, we will discuss insights into the impact of obesity on immune function gleaned from both genetically obese and DIO mice.

**Figure 4:**
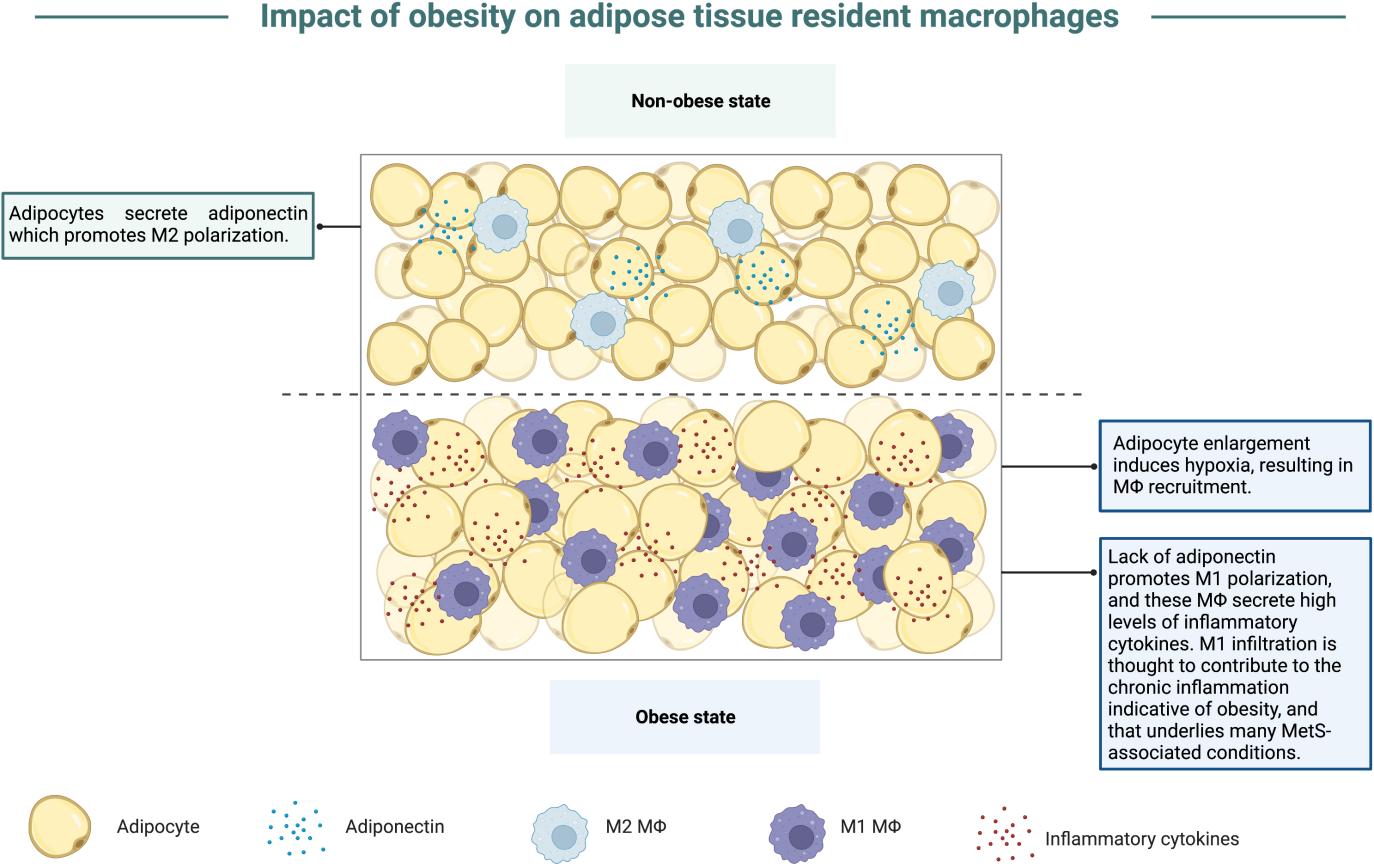
Obesity promotes accumulation of M1 macrophages. In the non-obese state, adipocytes secrete adiponectin, an adipokine that promotes macrophage polarization to the M2 phenotype. Within the obese state, adipocytes enlarge to store excess nutrients from overnutrition, which results in hypoxia and macrophage recruitment into adipose tissue. In the hypoxic state, less adiponectin is secreted, thus inducing macrophage polarization to the M1 phenotype, contributing to a state of chronic inflammation as these macrophages secrete high levels of inflammatory cytokines. This chronic inflammation is also believed to underlie and link the conditions that encompass MetS.

Animal studies interrogating the impact of obesity on viral disease severity and antigen-specific immune responses have repeatedly illustrated that obesity exacerbates viral disease severity and dampens virus-specific immune responses (14, 63–68). Studies have suggested that a major contributor to obesity-associated immune dysfunction is the skewing of macrophage polarization within excessive stores of adipose tissue. Nearly since the advent of murine obesity studies, macrophages have been noted at the forefront of obesity-associated immune dysregulation due to their accumulation in adipose tissue. Though previously thought to be a neutral storage site for excess lipids, adipose tissue is an endocrine organ that secretes an array of hormones and adipokines central to maintaining systemic metabolism (69). Although adipose tissue (AT) houses several resident cell types, the most abundant leukocyte population found here is adipose tissue-resident macrophages (ATM) (70). In the obese state, ATM populations greatly increase in humans and mice, at times constituting 50% of the tissue (71). This intense inflation of ATM numbers in the obese state is believed to occur in part due to fat accumulation causing adipocytes to rapidly enlarge, thus inducing a hypoxic state which promotes necrosis and attracts more macrophages into the adipose tissue (reviewed in (72)). The increase in ATM, contributes to chronic low-grade inflammation which occurs due to the propensity for ATM to secrete large amounts of pro-inflammatory cytokines like TNF-α and interleukin-1β (IL-1β) (70, 73–76), graphically depicted in **Figure 4**. Numerous studies discussed below expand from focusing on macrophage-associated enhanced inflammation to detailing a role for obesity-induced defective type I interferon (IFN) responses. Many viral pathogens excel at antagonizing the host type I IFN signaling pathway to promote the production of progeny virions; thus, viral infection could be more fraught for individuals with obesity who may already have a blunted type I IFN response due to metabolic perturbances.

**Figure 5:**
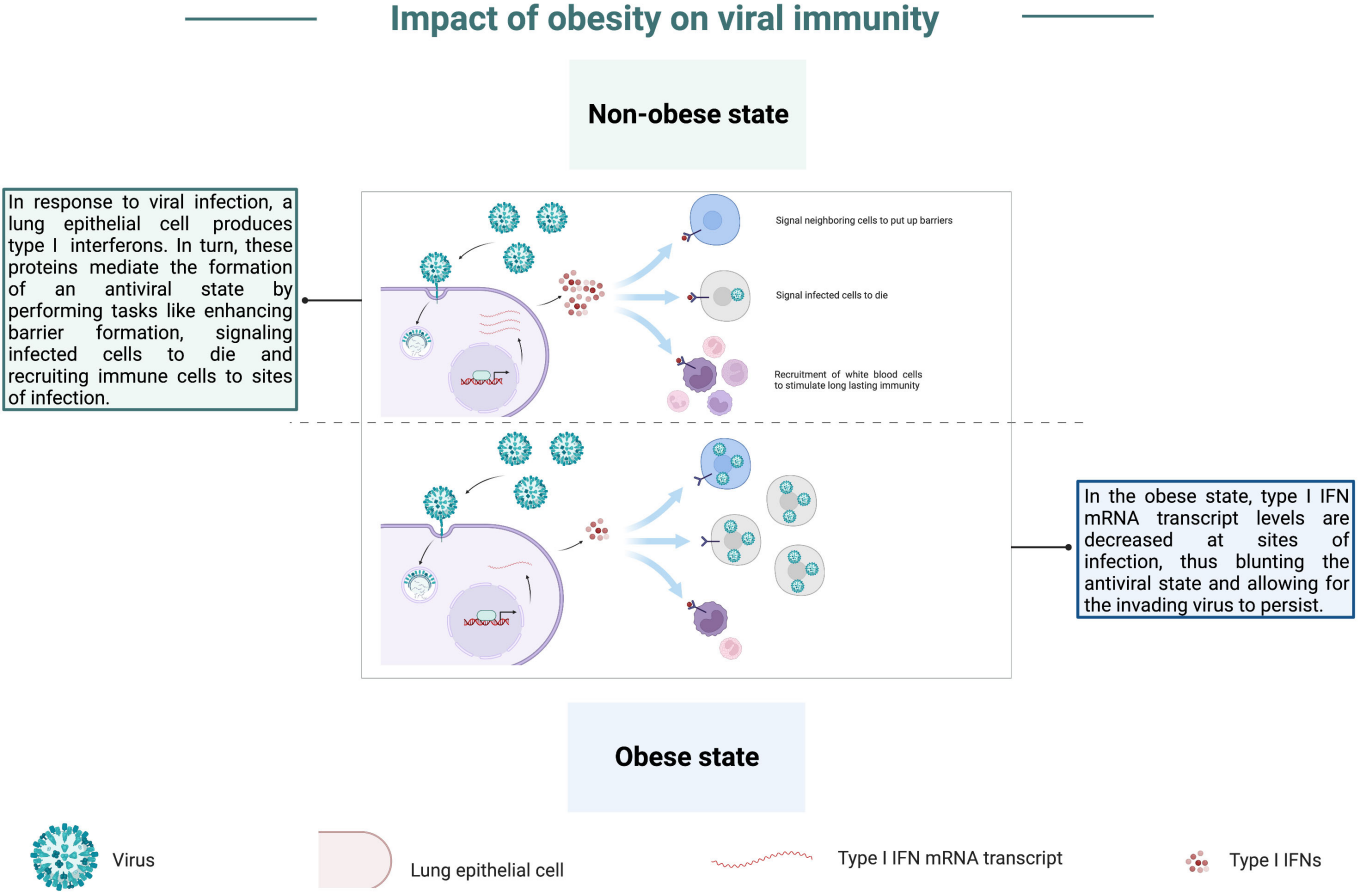
Obesity blunts anti-viral type I IFN responses. In the non-obese state, a lung epithelial cell produces type I interferons (type I IFNs) in response to infection by a respiratory pathogen. In turn, these proteins mediate the induction of an antiviral state through tasks such as enhancing barriers, signaling infected cells to die, and recruiting immune cells to infection sites. In the obese state, mRNA transcripts of type I IFNs are decreased at infection sites, thus blunting the induction of an antiviral state and allowing the invading virus to persist.

Studies of the interplay between obesity and viral immunity have focused heavily on respiratory pathogens, such as influenza virus, particularly in the aftermath of the 2009 H1N1 pandemic. In several studies, obese mice displayed significantly higher mortality rates compared to wild-type counterparts following influenza virus infection (66, 77–81), and reducing the dose of influenza virus given to obese animals was insufficient for mitigating their enhanced mortality (64). Obese mice often showed higher lung titers (66, 67, 80), in addition to elevated lung inflammation, leading to more significant pathology and tissue damage (67, 77–81). In an experimental study, O’Brien et al. observed significantly higher host and viral protein levels in bronchoalveolar lavages from obese animals, suggesting obese mice experienced defects in the maintenance of the barrier permeability (81), thus potentially accounting for enhanced edema and inflammation seen in these animals post-influenza virus infection. Interestingly, elevated lung titers are not the only factor contributing to enhanced respiratory tract pathology, as obese mice in a study done by Milner et al. had an equivalent viral burden in the lungs when compared to wild-type mice, yet significantly greater levels of lung inflammation and tissue damage (79). Similarly, elevated lung viral titers cannot be attributed as the sole cause for enhanced mortality as O’Brien et al. found enhanced mortality and inflammation in obese animals when compared to wild-type counterparts, yet obese animals in this study did not display significantly higher viral titers or worsened pathology in comparison to wild-type animals (81). Potentially underlying these contradictory findings are studies aimed at characterizing expression of interferon α and β (IFN-α and IFN-β) at sites of infection within the respiratory tracts of obese mice. These type I IFN anti-viral cytokines are essential for establishing an anti-viral state, and their mRNA transcript levels were markedly lower in obese mice when compared to the expression noted in wild-type mice following influenza virus infection (66, 77, 78, 82), graphically depicted in **Figure 5**; the same was true for expression of pro-inflammatory cytokine- and chemokine-encoding mRNAs (77, 80, 81). These findings pin obesity at the forefront of inhibiting the innate immune system from adequately responding to acute viral infection. This type of immune defect not only poses a significant risk for combatting viral infections in the early stages of infection, where innate immune cells mount a rapid, multifaceted anti-viral attack, but also a blunted or delayed innate immune response could have detrimental consequences that prevent the priming of a robust adaptive immune response. In further examples of the propensity for the obese state to blunt type I IFN responses, Honce et al. noted that viral variants were detected early in obese mice post-influenza virus inoculation relative to wild-type mice (63). These variants replicated quickly within the obese hosts and exhibited greater virulence compared to the parental infecting strain (63). It is possible that these variants arose specifically within obese mice due to their blunted, delayed type I IFN response. This finding provides additional insight into why obesity has been linked to higher morbidity and mortality post-viral infection.

Moreover, confirming the impact obesity has on mortality following viral infection, Karlsson et al. sought to determine if obesity impacted memory T cell responses, thus predisposing hosts for greater susceptibility to severe viral disease following infection with a previously encountered pathogen. As memory T cells primed during a primary influenza virus infection are specific for internal viral proteins typically shared among various influenza strains, these T cells are effective at combatting infection by heterologous strains. However, this study revealed that obese mice had a significantly higher mortality rate following secondary challenge with a different influenza strain when compared to survival rates of wild-type counterparts (66). This study brings to the forefront the impact obesity can have on memory immune responses, as obese mice were not protected against a second encounter with an influenza virus. Following a similar line of questioning, wild-type and obese mice in another study were infected with sublethal doses of influenza virus, followed by a sublethal dose of *Streptococcus pneumoniae* (*S. pneumoniae)* (83). Obese animals succumbed to coinfection uniformly and significantly earlier than wild-type mice, and obese mice also displayed high viral and bacterial titers that correlated to extensive cellular damage (83). Interestingly, O’Brien et al. noted significantly less epithelial regeneration within bronchoalveolar surfaces when compared to wild-type mice (81), suggesting impaired wound healing in obese animals. This respiratory barrier vulnerability could account for their enhanced susceptibility to secondary infection. These studies reveal how, in addition to enhancing morbidity and mortality rates following a primary infection, obesity can also enhance host susceptibility to secondary infection, whether in the form of a challenge with the original invading pathogen, or a heterologous challenge with a different pathogen. These findings are critical to note as secondary infections are common among patients with influenza virus (84, 85) or SARS-CoV-2 (86–88) infections, confirming the toll obesity imposes on public health outcomes.

Finally, in addition to studies exploring the impact of obesity on respiratory infections, other research efforts have confirmed that obesity enhances morbidity and mortality in non-respiratory infections. In our previous study, we showed that dengue virus infection caused enhanced morbidity in obese mice based on weight loss and thrombocytopenia compared to wild-type mice (89). Similarly, in a study examining the impact of obesity on alphavirus infection outcomes, we showed that obese mice displayed significantly higher morbidity in terms of weight loss and mortality following infection with Mayaro virus, chikungunya virus, and Ross River virus (90), all of which are mosquito-borne pathogens. Further, in our studies of West Nile virus (WNV), we noted that obese mice displayed significantly higher viral titers in peripheral organs and the brain. We also found that these mice had a significantly higher mortality rate post-WNV infection when compared to wild-type counterparts (68). Similarly, upon infection with the rodent-borne viral pathogen lymphocytic choriomeningitis virus (LCMV), obese mice again experienced significantly higher mortality rates compared to wild-type mice and elevated viral titers (91). Interestingly, Pepin et al. highlight an important finding utilizing antiretroviral therapy (ART) to manage human immunodeficiency virus (HIV) infection. Rather than directly interrogating the impact of obesity on HIV-associated immune defects, these authors draw attention to the inherent predisposition for metabolic derangements that individuals who rely on ART for managing HIV infections experience. For example, prolonged ART treatment does not restore proper immune function in patients with HIV, but rather it is typically associated with premature immune aging, persistent immune hyper-activation, and chronic inflammation (92). Unsurprisingly, these predispositions result in patients with HIV exhibiting impaired metabolic control (93), MetS-associated comorbidities like obesity (94), NAFLD (95), type 2 diabetes (96), and high prevalence of insulin resistance (97–99). In their study, these authors found that ART worsened high fat diet-induced MetS conditions in mice, like enhanced glucose dysregulation (100). Further, ART exaggerated adiposity in the obese mice, and contributed to further macrophage infiltration and polarization to the M1 phenotype, accompanied by enhanced inflammation (100). Taken together, these data highlight the capability of obesity to predispose for heightened viral disease severity and mortality to an array of pathogens with varying tropisms, and some antiviral treatments can compound the impacts of obesity on antiviral immunity, thus increasing the burden of viral diseases in humans with metabolic derangements.

## Impact of obesity on vaccine efficacy

As a final comment on the impact of obesity on overall viral immunity, it is important to consider vaccine efficacy amidst a state of metabolic perturbances. There have been many recent outstanding reviews covering the clinical studies that demonstrate a reduced COVID-19 and IAV vaccine durability (101–104). Additionally, hospitalizations due to SARS-CoV-2 breakthrough infections are significantly elevated in individuals with type 2 diabetes, cardiovascular disease, as well as in individuals who are overweight (105–107). Similar to the viral infection studies discussed above, previous studies in animals have noted that metabolic dysfunction, and obesity in particular, are predictors of poor vaccine responses. Karlsson et al. conducted a study that eloquently demonstrated the negative impact obesity can have on vaccine-conferred immunity. In this study, wild-type and obese mice were infected with a nonlethal dose of influenza virus followed by a nonlethal dose of *S. pneumoniae* to simulate a secondary bacterial infection, a common risk factor associated with influenza virus infections in vulnerable populations. As mentioned earlier in this review, coinfected obese mice displayed a significantly higher mortality rate when compared to coinfected wild-type counterparts (83). The authors of this study noted that vaccinating obese mice against either pathogen failed to protect these animals from heightened mortality. Highlighting that obesity prevented the production of a protective vaccine-induced adaptive immune response.

Providing insight into why the vaccines fail to confer protection in obese animals, other studies revealed impaired vaccine-induced immune responses in obese animal (64, 67, 80, 108–110). Specifically, the frequency of antigen-specific CD8 T cells found in obese mice following influenza virus vaccination was significantly lower than in wild-type counterparts (67). Further, Karlsson et al. noted a reduction in the expression of interleukin-7 (IL-7) on antigen-specific CD8 T cells following influenza virus infection, and this study, as well as others, revealed that obese-primed T cells exhibited a steep decline in their ability to secrete IFN-γ when compared to wild type-primed CD8 T cells (65, 66, 111). This finding is consistent with studies done on IL-7 signaling-deficient mice following influenza virus infection where a decreased accumulation of antigen-specific, functionally active CD8 T cells existed at sites of infection (112). Taken together, these findings are informative for vaccine design, as IL-7 plays a canonical role in maintaining memory CD8 T cells (113), a key fact that makes data generated by Milner et al. and Rebeles et al. illuminating. Milner et al. found that influenza-specific CD8 T cells primed in obese mice produced less IFN-γ during a secondary exposure when compared to their cytokine production during a primary infection (67). Further, Rebeles et al. noted that upon a secondary influenza virus challenge, the number of CD8 T cells at sites of infection in obese mice were significantly reduced compared to the lungs of wild-type counterparts. They also noted that recalled CD8 T cells in obese mice exhibited altered cellular metabolism patterns characterized by increased oxygen consumption (108). These findings suggest that the obese microenvironment negatively impacts the maintenance of memory CD8 T cells, thus dampening their ability to respond quickly and effectively upon antigen re-exposure; consequently, these phenomena could explain why vaccine efficacy appears to be reduced in hosts with obesity.

Pursuing this matter further, several studies reported that obesity leads to reduced vaccine-induced antibody production following vaccination in obese mice compared to wild-type counterparts (64, 67, 80, 109). This finding indicates that the obese environment fails to foster an expansive antibody repertoire which could account for higher susceptibility to severe viral disease upon secondary exposure. Coupled with lower overall antibody titers, non-neutralizing antibody levels also waned significantly faster in obese animals when compared to wild-type counterparts following vaccination (109). Similarly, serum antibody neutralization capacity was markedly reduced in obese mice following influenza vaccination (80, 110). Taken together, these data indicate that obesity dampens the generation of a robust antibody response post-vaccination, and highlights that antibodies generated in the obese state tend to wane more rapidly than those generated in a metabolically healthy microenvironment.

## Impact of hyperglycemia on viral immunity

Serving as another contributing factor to MetS diagnosis, this section will focus on hyperglycemia and insulin resistance. Hyperglycemia refers to a state where excess sugar (glucose) circulates in the blood. In a healthy physiological state, pancreatic beta cells produce insulin and release it into the bloodstream when circulating glucose levels are high. Insulin stimulates cells to capture glucose from the bloodstream, thus lowering blood glucose levels. When glucose levels in the blood remain high, beta cells are stimulated to secrete higher insulin levels to counteract hyperglycemia. An overabundance of insulin in the bloodstream can gradually desensitize cells to the protein, thus making them less likely to store circulating glucose, a phenomenon known as insulin resistance. Simultaneously, the increased effort exerted by beta cells to counteract chronic hyperglycemia can lead to cellular exhaustion, thus damaging the population of beta cells and inhibiting future insulin release (114). When insulin resistance occurs, causing blood glucose levels to remain high, this impairment in glucose storage and regulation is referred to as type 2 diabetes (115). Previously, urinary tract infections were the most reported immune system-related susceptibility for type 2 diabetes patients (116). However, data recorded from SARS-CoV-2 patients has pinned type 2 diabetes as a significant risk factor for predicting severe viral infection outcomes (117, 118). Throughout this section, we will discuss some of the data that have been reported regarding the impact of hyperglycemia and insulin resistance, or diabetes, on immunity to viral infections.

**Figure 6:**
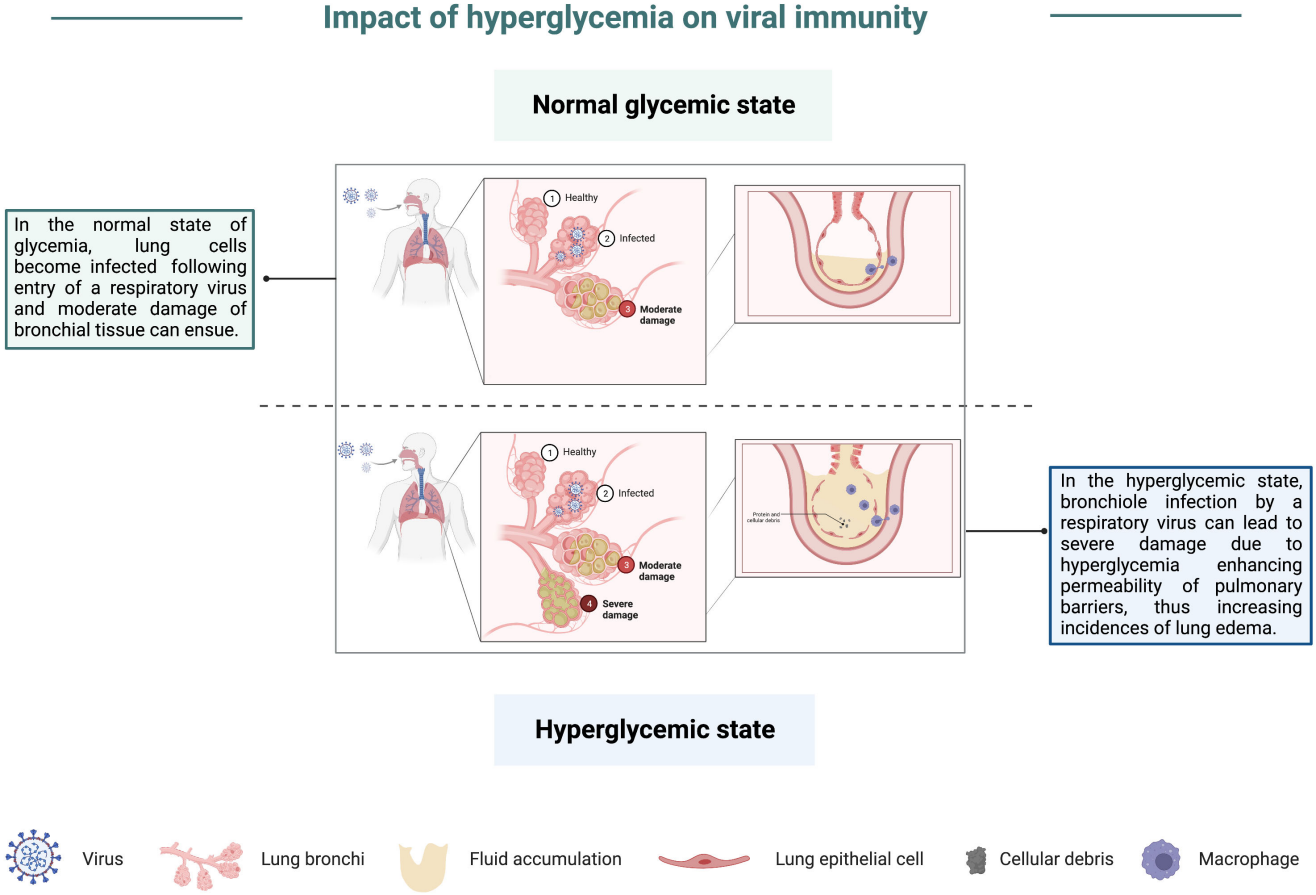
Impact of hyperglycemia on viral immunity. In a state of normal glycemia levels, lung cells can become infected by a respiratory virus, which may result in moderate damage to bronchial tissue. However, in the hyperglycemic state, severe damage of bronchioles following viral pathogen infection is more likely due to an impairment in lung barriers, thus enhancing their permeability and increasing the incidence of lung edema.

Historically, studies elucidating the link between viruses and hyperglycemia have focused on the role of viruses in inducing or exacerbating diabetes. These studies done both *in vitro* and *in vivo* in small animal models have utilized numerous viral pathogens, including herpes viruses (119), encephalomyocarditis virus (EMCV) (120, 121), coxsackievirus B4 (122), and reoviruses (123). Infection by the viruses highlighted in these studies was shown to inflict cellular damage on beta cells, leading to hypoinsulinemia, and consequently causing hyperglycemia. While not directly demonstrating how viral disease severity is enhanced in instances of hyperglycemia, these studies have provided an ideal model of understanding the relationship between hyperglycemia and shortened lifespan, in addition to revealing an association between viral infection and blood glucose levels. Around 2000, clinical studies began to record an increase in the risk of severe viral disease in patients with hyperglycemia (124). By 2004, plasma glucose levels were shown to be a predictor of mortality following SARS-CoV infection (125, 126). Studies by Kumar et al. noted that WNV infection in diabetic mice led to more severe disease (127), similar to what has been observed in humans following WNV infection (128–131). IAV studies done with insulin receptor-deficient mice, which mimic human insulin resistance, demonstrated that insulin resistance resulted in reduced immune responses and poor protection against an H1N1 challenge (132). More recently, Hulme et al. found that mice with high glucose levels had increased disease severity following IAV infection. The investigators further demonstrated that elevated disease severity was due to hyperglycemia-induced damage to the pulmonary epithelial: endothelial barrier, thus increasing lung edema (133), graphically depicted in **Figure 6**. *In vitro* studies investigating mechanisms of impaired immune function associated with hyperglycemia in mice have noted that hyperglycemia can alter innate immune antiviral defenses (134), thus blunting early immune responses to viruses. Additionally, in studies looking at immune cell differentiation in hyperglycemic mice, authors noted that hyperglycemia alters the differentiation of endothelial progenitor cells, thus leading to an increase in the frequency of proinflammatory cells (135). This predisposition of cells from hyperglycemic mice to differentiate into inflammatory mediators may contribute to the chronic inflammation associated with immune dysfunction and MetS, especially when considering that inflammatory cytokines, namely TNF-α, are insulin-desensitizing (5, 136, 137).

## Impact of hypertension on viral immunity

Hypertension, or high blood pressure, results when the force exerted by blood circulating against arterial walls within the body’s major blood vessels becomes elevated (138). Hypertension is the most common chronic disease condition in the world, and due to the common co-occurrence of hypertension with the previously described metabolic perturbance characteristics of MetS, a correlation between hypertension and altered immunity to viral pathogens would be unsurprising. However, whether high blood pressure alone can alter immunity is an active area of investigation, especially among cohort studies conducted on SARS-CoV-2 patients.

**Figure 7:**
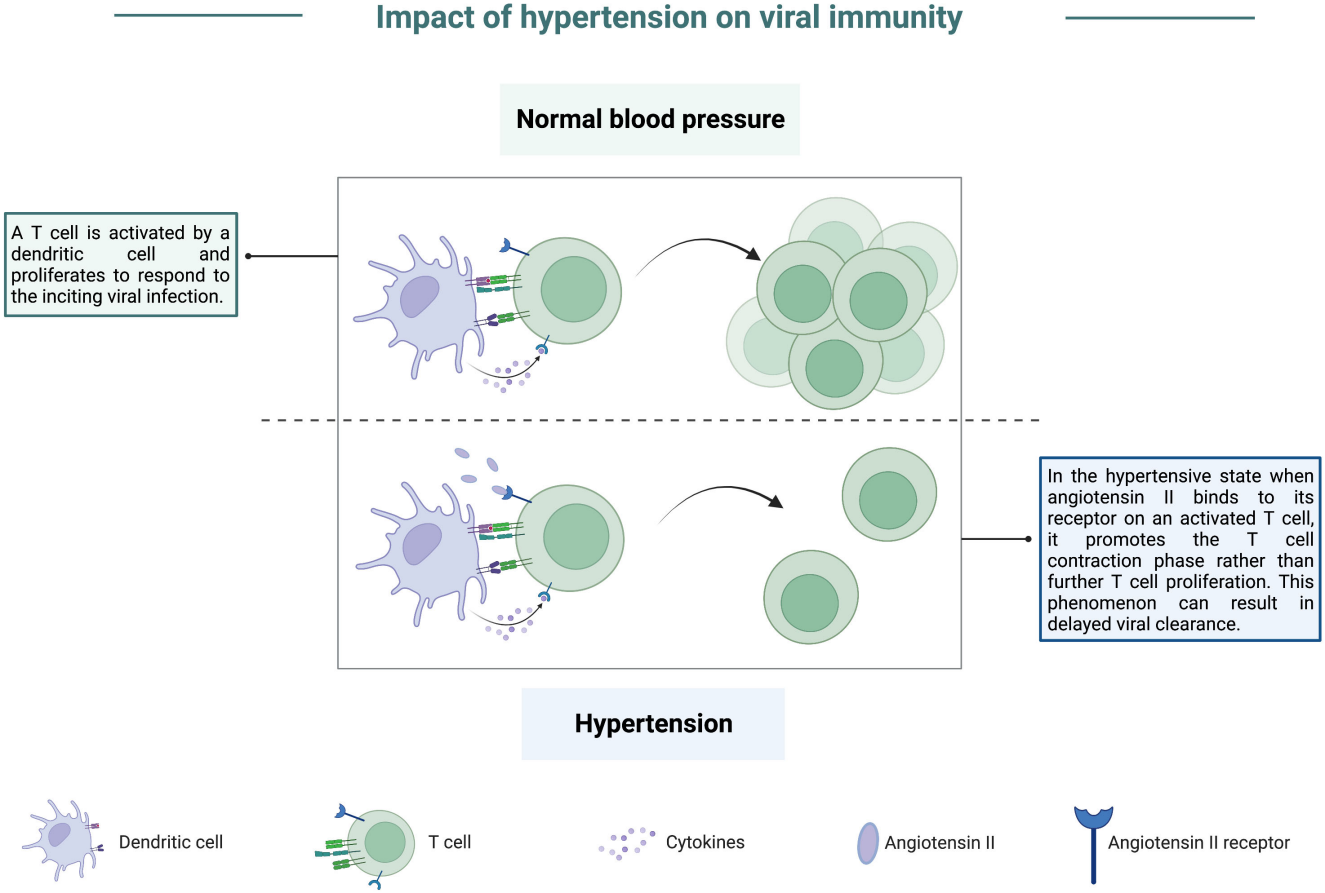
Impact of hypertension on viral immunity. In a state of normal blood pressure levels, T cells can be activated and proliferate to respond to virally infected cells following antigen presentation by a dendritic cell. However, in the hypertensive state, angiotensin II levels are higher, and this protein can bind to its receptor present on activated T cells. Upon binding, angiotensin II promotes the T cell contraction phase at the expense of sustaining a robust effector response. This phenomenon can result in delayed viral clearance due to a lack of T cells counteracting virally infected cells.

Shortly after the link between hypertension and viral disease was discovered, many groups worked to develop animal models that would mimic hypertension to develop treatments and provide a better understanding of the causes of this highly prevalent chronic condition (139). As with animal studies of hyperglycemia, early studies focused on viral infections which caused hypertension in both animals and humans, with some of the most notable studies looking at the role of cytomegaloviruses (CMV) in contributing to hypertension (140, 141). Although it has yet to be studied extensively in small animal models, data arising from the ongoing SARS-CoV-2 pandemic has identified hypertension as a factor that can contribute to severe COVID-19. Compared to COVID-19 patients with healthy blood pressure, hypertensive individuals were more likely to develop severe pneumonia or organ damage and experience a delay in viral clearance (142). Further, individuals with hypertension also displayed exacerbated inflammatory responses post-viral infection and had a heightened risk for mortality following SARS-CoV-2 infection (143, 144). Interestingly, individuals with a history of hypertension but currently being treated with anti-hypertensive medications had a substantial decrease in the likelihood of critical outcomes from COVID-19 (142). Of the minimal number of animal studies conducted to assess the impact of hypertension on susceptibility to viral infection, several have noted that sympathetic nerve activity is exaggerated (145, 146) and thought to contribute to enhanced morbidity and mortality in hypertensive patients (147). Specifically, angiotensin II (AngII) expression is elevated in the hypertensive state and can activate sympathetic nerves, increasing proinflammatory cytokine expression (148). This increase may account for enhanced inflammation noted in hypertensive COVID-19 patients and exacerbated organ damage. Interestingly, one of the receptors for AngII, angiotensin II type 1-receptor (AT1R), is expressed on T lymphocytes. Studies in murine models revealed that engagement of AngII with AT1R decreased the activation of antigen-specific CD8 T cells (149). This engagement also accelerated the contraction phase of T cells in response to stimulation by their antigen (149), graphically depicted in **Figure 7**. This finding is also enlightening in the context of hypertensive COVID-19 patients as it could partially explain the delayed viral clearance observed in these patients.

## Conclusions

As highlighted in this review, human cohort studies of various metabolic perturbances associated with MetS have drawn attention to the impact of aberrant metabolism on viral disease severity and vaccination outcomes. Serving as essential tools for elucidating the effect of specific physiological perturbances, small animal models have allowed scientists to begin understanding how conditions such as obesity, high cholesterol, hypertriglyceridemia, hyperglycemia, and hypertension induce aberrant immunity to viral pathogens. These findings suggest that the development of MetS leads to a blunted host immune response to viral infection by influencing the immune system in different ways, meaning that patients with MetS who often co-present with these inherently overlapping risk factors could be at even higher risk of severe viral disease development than patients harboring one of these conditions independently. Future small animal model studies centered on exploring the interplay between MetS and viral immunity or vaccination are of great importance as the proportion of individuals diagnosed with MetS is projected to rise continually.

## Author contributions

All listed authors have directly contributed to the conception of this review and have read and approved the submitted article.

## Funding

This effort was supported by a Discovery award USAMRDCPR192269 from Department of Defense.

## Conflict of interest

The authors declare that the research was conducted in the absence of any commercial or financial relationships that could be construed as a potential conflict of interest.

## Publisher’s note

All claims expressed in this article are solely those of the authors and do not necessarily represent those of their affiliated organizations, or those of the publisher, the editors and the reviewers. Any product that may be evaluated in this article, or claim that may be made by its manufacturer, is not guaranteed or endorsed by the publisher.
